# Supplementary material for: The Malay-Version Knowledge, Risk Perception, Attitude and Practice Questionnaire on Heatwaves: Development and Construct Validation
Source: Int J Environ Res Public Health. 2022 Feb 17;19(4):2279. doi: 10.3390/ijerph19042279 (PMC8872578; doi:10.3390/ijerph19042279)
Supplement: Supplementary file 1 [file ijerph-19-02279-s001.zip › Supplementary file S1. Details KRPAP constructs.pdf]

Supplementary File S1. Details of objectives and corresponding items and statements of knowledge, risk perception, attitude, and practice constructs

CONSTRUCT	OBJECTIVE	CODE	STATEMENTS
Knowledge<br><br>*(Pengetahuan)*	Measure the level of knowledge regarding heatwaves<br><br>*(Mengukur tahap pengetahuan berkaitan gelombang haba)*	K1*	The definition of heat wave in Malaysia is the maximum temperature between 35.0 ° C - 37.0 ° C for three consecutive days<br><br>*Definisi gelombang haba di Malaysia adalah suhu maksimum antara 35.0°C - 37.0°C selama tiga hari berturut-turut*
		K2*	Heat waves are caused by high temperatures and less rainfall distribution<br><br>*Gelombang haba disebabkan oleh suhu yang tinggi dan taburan hujan yang kurang*
		K3*	The Department of Environment is responsible for reporting heat wave incidents in Malaysia<br><br>*Jabatan Alam Sekitar bertanggungjawab melaporkan kejadian gelombang haba di Malaysia*
		K4*	The occurrence of heat waves will increase the temperature of the human body<br><br>*Kejadian gelombang haba akan meningkatkan suhu badan manusia*
		K5*	Suhu badan normal manusia adalah 36.5°C - 37.5°C<br><br>*Normal human body temperature is 36.5 ° C - 37.5 ° C*
		K6*	The period of time required by the human body to adapt to hot temperatures is around two hours to 1 day<br><br>*Tempoh masa yang diperlukan oleh tubuh badan manusia untuk menyesuaikan diri dengan suhu panas adalah sekitar dua jam hingga 1 hari*
		K7*	Among the signs of heat stress are profuse sweating, nausea, and headaches<br><br>*Antara tanda tekanan haba adalah berpeluh dengan banyak, mual, dan sakit kepala*
		K8	Diabetes is an example of a disease caused by extreme heat<br><br>*Kencing manis ialah contoh penyakit yang dihidapi akibat panas terik melampau*

		K9*	Heat waves can be a factor in the occurrence of depression<br><br>*Gelombang haba boleh menjadi faktor kepada terjadinya kemurungan*
		K10*	Heat -related illnesses can lead to death<br><br>*Penyakit berkaitan kepanasan haba boleh membawa kepada kematian*
		K11*	Individuals with heart problems are more likely to get sick during heat waves<br><br>*Individu yang mempunyai masalah jantung lebih berkemungkinan untuk jatuh sakit sewaktu gelombang haba*
		K12*	Children are more at risk of getting sick during heat waves<br><br>*Golongan kanak-kanak adalah lebih berisiko untuk jatuh sakit semasa gelombang haba*
		K13*	The elderly (65 years and older) are more at risk for getting sick during heat waves<br><br>*Golongan orang tua (65 tahun dan ke atas) adalah lebih berisiko untuk jatuh sakit semasa gelombang haba*
		K14*	Those who work outside the field are less at risk of getting sick during heat waves<br><br>*Golongan yang bekerja di luar lapangan adalah kurang berisiko untuk jatuh sakit semasa gelombang haba*
		K15*	The use of a hat or umbrella when outdoors can reduce the risk of heatstroke<br>*Penggunaan topi atau payung ketika berada di luar dapat mengurangkan risiko strok haba*
		K16*	Using a sunscreen can reduce the risk of heat stroke<br><br>*Menggunakan krim pelindung matahari dapat mengurangkan risiko strok haba*
		K17	Wearing dark-colored clothes can help cool the body during heat waves<br><br>*Pemakaian baju berwarna gelap dapat membantu menyejukkan badan semasa gelombang haba*
		K18	Doing physical activity regularly during heatwaves can avoid the effects of heatwaves<br><br>*Melakukan aktiviti fizikal dengan kerap semasa gelombang haba dapat menghindari diri daripada kesan gelombang haba*
		K19*	Heatwaves can cause forest fires

			Gelombang haba boleh menyebabkan berlakunya kebakaran hutan
		K20	Heatwaves should not cause damage to coral reefs<br><br>*Gelombang haba tidak boleh menyebabkan kerosakan terumbu karang*
Risk Perception<br><br>*(Persepsi Risiko)*	Measure the perceived threat from heat waves<br><br>*(Mengukur ancaman yang dirasakan dari gelombang haba)*	RP1	I will experience loss of body fluids (dehydration) during heat waves<br><br>*Saya akan mengalami kehilangan cecair badan (dehidrasi) semasa gelombang haba*
		RP2*	My body temperature will rise dramatically during heat waves<br><br>*Suhu badan saya akan meningkat dengan mendadak semasa gelombang haba*
		RP3*	My body will experience fatigue during heat waves<br><br>*Badan saya akan mengalami keletihan semasa gelombang haba*
		RP4*	If I am exposed to heat waves for too long, I will get sunburn<br><br>*Jika terlalu lama terdedah kepada gelombang haba, saya akan mengalami selar matahari (lecuran matahari)*
		RP5	I would experience depression during heat waves<br><br>*Saya akan mengalami kemurungan semasa gelombang haba*
		RP6	If my body temperature rises during heat waves, I may need to see a doctor<br><br>*Jika suhu badan saya meningkat semasa gelombang haba, saya mungkin perlu berjumpa dengan doktor*
		RP7*	If I experience loss of body fluids (dehydration) during heat waves, I may need to be hospitalized<br><br>*Jika saya mengalami kehilangan cecair badan (dehidrasi) semasa gelombang haba, saya mungkin perlu dimasukkan ke hospital*
		RP8*	Loss of body fluids (dehydration) during heat waves will affect my health for a long time<br><br>*Kehilangan cecair badan (dehidrasi) semasa gelombang haba akan menjejaskan kesihatan saya untuk jangka masa panjang*
		RP9	If I get a sunburn during heat waves, it can cause skin cancer

			Jika saya terkena selar matahari (lecuran matahari) semasa gelombang haba, ia boleh menyebabkan kanser kulit
		RP10*	If I get a sunburn during heat waves, it can cause skin cancer<br><br>*Saya tidak dapat bekerja sekiranya dimasukkan ke hospital akibat kehilangan cecair badan (dehidrasi) semasa gelombang haba*
Practice<br><br>*(Amalan)*	Measure respondents practices to avoid the effects of heatwaves<br><br>*(Mengukur amalan responden untuk mengelakkan kesan gelombang haba)*	P1	Eating hot foods will help me cope with heat waves<br><br>*Makan makanan yang panas akan membantu saya menghadapi gelombang haba*
		P2*	Being in a room with air conditioning will reduce my chances of dehydration<br><br>*Berada dalam ruang dengan penghawa dingin akan mengurangkan kemungkinan saya diserang kekurangan cecair badan (dehidrasi)*
		P3	By using sunscreen, I will be spared from sunburn during heat waves<br><br>*Dengan menggunakan krim pelindung matahari, saya akan terhindar daripada selar matahari (lecuran matahari) semasa gelombang haba*
		P4	Keeping up with the weather forecast on a daily basis will help me plan outdoor activities<br><br>*Dengan mengikuti perkembangan ramalan cuaca setiap hari akan membantu saya merancang aktiviti luar*
		P5*	Drinking lots of water to stay hydrated will help me cope with heat waves<br><br>*Minum banyak air utk kekal terhidrasi akan membantu saya menghadapi gelombang haba*
		P6*	Reducing outdoor activities and staying indoors will help me cope with heat waves<br><br>*Mengurangkan aktiviti luar dan kekal berada di dalam rumah akan membantu saya menghadapi gelombang haba*
		P7	Using a hat or umbrella when out of the house can prevent me from getting sick from heat waves<br><br>*Menggunakan topi atau payung semasa keluar rumah dapat mengelakkan diri saya daripada jatuh sakit akibat gelombang haba*
		P8	Bathing regularly during heat waves will cause water wastage, and my house water bill will increase

			Mandi dengan kerap semasa gelombang haba akan menyebabkan pembaziran air dan bil air rumah saya akan meningkat
		P9*	Due to safety factors, I would not open doors and windows at night for air to enter during heat waves<br><br>*Kerana faktor keselamatan, saya tidak akan membuka pintu dan tingkap pada waktu malam untuk udara masuk semasa gelombang haba*
		P10*	I will not use the air conditioner during heat waves as it will cause the cost of the electricity bill to increase<br><br>*Saya tidak akan menggunakan alat penghawa dingin semasa gelombang haba kerana akan menyebabkan kos bil elektrik meningkat*
		P11*	Due to the busy work factor, I could not rest and drink enough water during the heat wave<br><br>*Kerana faktor pekerjaan yang sibuk, saya tidak dapat berehat dan minum air secukupnya semasa terjadi gelombang haba*
		P12	I could not find out more information about heat waves because I did not have internet and social media resources<br><br>*Saya tidak dapat mengetahui maklumat lanjut berkenaan gelombang haba kerana tidak mempunyai sumber internet dan media sosial*
Attitude<br><br>*(Sikap)*	Measure respondents' attitudes to act to protect themselves from heat waves<br><br>*(Mengukur sikap responden untuk bertindak melindungi diri daripada gelombang haba)*	A1	Family members or friends told me about the dangers of heatwaves to the body<br><br>*Ahli keluarga atau rakan memberitahu saya tentang bahaya gelombang haba kepada tubuh badan*
		A2	I watched television about hospitalized patients due to lack of body fluids (dehydration) during heat waves<br><br>*Saya menonton televisyen berkenaan pesakit dimasukkan ke hospital akibat kekurangan cecair badan (dehidrasi) sewaktu gelombang haba*
		A3*	I read information about the dangers of heat waves to health in local newspapers, television and social media<br><br>*Saya membaca maklumat tentang bahaya gelombang haba terhadap kesihatan di akhbar tempatan, televisyen dan media sosial*

		A4*	The doctor reminded me of the dangers of heat waves<br><br>*Doktor ada mengingatkan saya tentang bahaya gelombang haba*
		A5*	I got information from the government agencies regarding the dangers of heatwaves<br><br>*Saya mendapat maklumat daripada agensi kerajaan berkenaan bahaya gelombang haba*

*finalised items
