# Supplementary material for: The Malay-Version Knowledge, Risk Perception, Attitude and Practice Questionnaire on Heatwaves: Development and Construct Validation
Source: Int J Environ Res Public Health. 2022 Feb 17;19(4):2279. doi: 10.3390/ijerph19042279 (PMC8872578; doi:10.3390/ijerph19042279)
Supplement: Supplementary file 1 [file ijerph-19-02279-s001.zip › Supplementary file S2. Summary of Model Fit Indices.pdf]

Supplementary File S2. Summary of Model Fit Indices

FIT INDICES	VALUE		CUT-OFF VALUE	REFERENCES
	Model 1	Model 2		
Chi square /degree of freedom	1.803	1.557	< 5.0<br>< 5.0 if N>200<br>< 3.0	(Marsh & Hocevar 1985) [40]<br>(Bentler 1990) [41]<br>(Hair et al. 2010) [46]
Comparative Fit Index (CFI)	0.913	0.942	> 0.9<br>> 0.9	(Bentler 1990) [41]<br>(Hatcher 1994) [43]
Goodness of Fit Index (GFI)	0.883	0.902	> 0.9	(Chau 1997) [42]
Incremental Fit Index (IFI)	0.914	0.943	> 0.9	(Hatcher 1994) [43]
Parsimony-Normed Fit Index (PNFI)	0.695	0.692	> 0.5	(Hatcher 1994) [43]
Root Mean Square Error of Approximation (RMSEA)	0.070	0.058	< 0.08	(Byrne 2001) [44]
