# Supplementary material for: The Malay-Version Knowledge, Risk Perception, Attitude and Practice Questionnaire on Heatwaves: Development and Construct Validation
Source: Int J Environ Res Public Health. 2022 Feb 17;19(4):2279. doi: 10.3390/ijerph19042279 (PMC8872578; doi:10.3390/ijerph19042279)
Supplement: Supplementary file 1 [file ijerph-19-02279-s001.zip › Supplementary file S3. Construct convergent and discriminant validity.pdf]

Supplementary File S3. Construct/convergent and discriminant validity

VALIDITY	CONSTRUCT	MODEL 2 (BASELINE)			MODEL 3 (A2 REMOVED)		
		**CR**	**AVE**	**MSV**	**CR**	**AVE**	**MSV**
Construct/Convergent	Practice	0.828	0.453	0.350	0.828	0.453	0.350
	Risk perception	0.815	0.432	0.350	0.815	0.432	0.350
	Attitude	0.784	0.479	0.076	0.776	0.536	0.073
		Practice	Risk perception	Attitude	Practice	Risk perception	Attitude
Discriminant	Practice	**0.673**			**0.673**		
	Risk perception	0.538	**0.657**		0.538	**0.657**	
	Attitude	0.181	0.356	**0.692**	0.138	0.331	**0.732**
	Heterotrait-Monotrait (HTMT) Warnings	No			No		

CR: Composite Reliability; AVE: Average Variance Extracted; MSV: Maximum Shared Variance
